# Induction of Early Autophagic Process on *Leishmania amazonensis* by Synergistic Effect of Miltefosine and Innovative Semi-synthetic Thiosemicarbazone

**DOI:** 10.3389/fmicb.2017.00255

**Published:** 2017-02-21

**Authors:** Débora B. Scariot, Elizandra A. Britta, Amanda L. Moreira, Hugo Falzirolli, Cleuza C. Silva, Tânia Ueda-Nakamura, Benedito P. Dias-Filho, Celso V. Nakamura

**Affiliations:** ^1^Laboratório de Inovação Tecnológica no Desenvolvimento de Fármacos e Cosméticos, Departamento de Farmácia, Universidade Estadual de MaringáMaringá, Brazil; ^2^Departamento de Química, Universidade Estadual de MaringáMaringá, Brazil

**Keywords:** *Leishmania amazonensis*, drug combination, apoptosis, autophagy, synergism, miltefosine, thiosemicarbazone

## Abstract

Drug combination therapy is a current trend to treat complex diseases. Many benefits are expected from this strategy, such as cytotoxicity decrease, retardation of resistant strains development, and activity increment. This study evaluated *in vitro* combination between an innovative thiosemicarbazone molecule – BZTS with miltefosine, a drug already consolidated in the leishmaniasis treatment, against *Leishmania amazonensis*. Cytotoxicity effects were also evaluated on macrophages and erythrocytes. Synergistic antileishmania effect and antagonist cytotoxicity were revealed from this combination therapy. Mechanisms of action assays were performed in order to investigate the main cell pathways induced by this treatment. Mitochondrial dysfunction generated a significant increase of reactive oxygen and nitrogen species production, causing severe cell injuries and promoting intense autophagy process and consequent apoptosis cell death. However, this phenomenon was not strong enough to promote dead in mammalian cell, providing the potential selective effect of the tested combination for the protozoa. Thus, the results confirmed that drugs involved in distinct metabolic routes are promising agents for drug combination therapy, promoting a synergistic effect.

## Introduction

Drug combination therapy is widely used to treat diseases resistant to conventional therapy, such as cancer and AIDS. Currently, this strategy have been employed to avoid or delay the development of resistant mechanism. Other benefit reached by drug association therapy is the toxicity reduction, since lower doses of each combined drug is at least enough to obtain the same effect of drugs singly. However, the synergistic effect should go beyond the sum of the individual effects (Chou, 2006). The traditional use of medicinal plants in the treatment of several diseases is also based on the synergistic effect from molecules in the complex matrix, commonly found in small quantities in plants and inactive by themselves. Synergistic effect could occur by modifying the stability, bioavailability, solubility, and half-life of pharmacologically active substance (Bone and Mills, 2013).

Thiosemicarbazones derivatives have been exhibited a wide spectrum biological activity, such as antibacterial (Viñuelas-Zahínos et al., 2011), antiprotozoa (Chellan et al., 2010; Britta et al., 2012, 2014; Fernández et al., 2015); and antitumor (Ferraz et al., 2011). Triapine^®^, the most famous thiosemicarbazone, have been studied in clinical trials for treat different diseases using distinct strategies, including the combination with another drug, according to U.S. National Institutes of Health^1^. Previously, 4-nitro benzaldehyde thiosemicarbazone derived from S-(-)-limonene (BZTS) revealed promising *in vitro* antileishmania activity (Britta et al., 2014).

Leishmaniasis represents a serious health public problem and is endemic in 88 countries, mainly tropical and underdeveloped places, where ideal climate conditions are found to sandfly vector development. Thirty thousand deaths occur annually in consequence of the main clinical manifestations of leishmaniasis (World Health Organization, 2016). Pentavalent antimonials are the first choice medicine against *Leishmania* spp. since 1940s. Severe side effects and increasing reports of resistant strains make the search for new drugs and development of new therapeutic strategies an urgent need from the clinical point of view (Georgiev, 1997; Lira et al., 1999; Bahreini et al., 2015).

Amphotericin B and miltefosine (or hexadecyl-phosphocholine), antifungal and antitumor originally drugs, respectively, have been used as second line drugs to treat leishmaniasis (Berman, 1997). Side effects, including teratogenicity, nephrotoxicity, anemia, and cardiotoxicity, are responsible for the difficulty to perform the complete treatment (Dorlo et al., 2012). As consequence, resistant strains development have been reported for all available drugs. Strategies to improve therapies using these medicines already consolidate at medical clinical are an interesting way to obtain advances in the leishmaniasis treatment.

Based in these informations and considering the interesting antileishmania profile of the BZTS, the main aim in this study was to analyze the combination of BZTS with amphotericin B and BZTS with miltefosine, evaluating antileishmania activity, cytotoxicity, and the death mechanism induced from drug combination.

## Materials and Methods

### Chemical Synthesis

4-Nitrobenzaldehyde thiosemicarbazone derived from S-(-)-limonene is an innovative substance synthesized at the State University of Maringa, according to Britta et al. (2014).

### Parasites and Macrophage Maintenance

*Leishmania amazonensis* MHOM/BR/75/Josefa strain was isolated from a human case of cutaneous leishmaniasis by Dr. Cesar A. Cuba, at University of Brasilia, Brazil (Saraiva et al., 1983; Cuba et al., 1985). These promastigote parasites were maintained in Warren’s medium, consisting of brain heart infusion (Gibco^®^), hemin (Sigma–Aldrich^®^), and folic acid (Sigma–Aldrich^®^), pH 7.2, supplemented with 10% of fetal calf serum (FCS – Invitrogen^®^) and incubated at 25°C.

Axenic amastigote forms were obtained from *L. amazonensis* promastigotes. For this, promastigote forms in phase log of growth were cultivated in Warren’s medium, pH 7.2, supplemented with 20% FCS and incubated at 25°C, during 48 h. Next, the parasites were maintained at 30°C, for 24 h, and at 32°C until differentiation in amastigote forms. Axenic amastigotes were maintained in Schneider insect’s medium (Sigma–Aldrich^®^), pH 4.6, supplemented with 20% of FCS, at 32°C (Ueda-Nakamura et al., 2001).

Murine monocytic lineage macrophages J774.A1 (Rio de Janeiro Cell Bank – BCRJ) were maintained in RPMI 1640 medium (Roswell Park memory Institute – Gibco^®^), pH 7.6, supplemented with 10% of FCS and 0.4% of penicillin-streptomycin antibiotics (Sigma-Aldrich^®^), incubated at 37°C, 5% CO_2_ tension and humidified atmosphere.

### *In vitro* Activity against *L. amazonensis*

Suspension of 1 × 10^6^ promastigotes/mL, in log phase of growth, was placed in 24-wells microplate in the presence of increasing concentrations of the BZTS, amphotericin B (Sigma–Aldrich^®^) and miltefosine (Avanti Polar Lipids Inc.), after previous solubilization with 0.5% DMSO (Synth^®^). The same procedure was carried out with axenic amastigote forms, using 12-wells microplate. After incubation during 72 h, at 25 and 32°C for promastigote and axenic amastigote forms, respectively, viable cells were counted with hemocytometer supporting. In order to obtain the IC_50,_ the concentration able to inhibit 50% of the growth compared to the control, the growth inhibition percentage was plotted in a graphic relating with the concentration of the compounds.

Furthermore, the activity of these drugs against *L. amazonensis* intracellular amastigotes was also evaluated. For this, intraperitoneal macrophages were harvested from the males BALB/c mice, by injection of cold phosphate-saline buffer (PBS) plus FCS 3% inner the peritoneal cavity. After centrifugation, a suspension of 5 × 10^5^ macrophages in RPMI was placed on glass coverslips in 24-wells microplate and incubated during 2 h, at 37°C and 5% CO_2_ atmosphere, for cellular adhesion. After this period, the wells were washed and the macrophages were infected with *L. amazonensis* promastigotes in stationary phase (5–6 days) at a ratio of 7 parasites/1 mammalian cell, during 4 h, at 34°C and 5% CO_2_ tension and then, the tested compounds were added during 48 h (Kaplum et al., 2016). At the end of this period, the glass coverslips were subject to fixation with methanol during 10 min and stained with 10% Giemsa stain (GIBCO KaryoMax^®^ Giemsa), for 40 min. Then, a total of 200 macrophages were counted under light microscope, distinguishing infected cells and amastigote parasites. Thereby, the survival index (*SI = infected cells percentage × amastigote average per infected macrophage*) was calculated considering 100% of survival for the untreated negative control (NC). It was subsequently possible to carry out the comparison with samples treated for IC_50_ calculation purposes.

### Ethics Statement

This study was approved and performed in accordance with the recommendations of Ethical Committee of animal use of Universidade Estadual de Maringá (protocol n° 2700110515/2015).

### Cytotoxicity and Hemolysis Assays

Cytotoxicity effects from isolated compounds were evaluated on J774.A1 lineage macrophages and applying colorimetric MTT method to measure the cell viability. For this, 5 × 10^5^ J774.A1 macrophages were seeded in 96-well microplate and incubated for 24 h, at 37°C and 5% CO_2_ to promote the multiplication to form a monolayer of confluent cells. Increasing concentrations of the BZTS, amphotericin B and miltefosine were added on the mammalian cells and incubated again under the same conditions for 48 h. In order to evaluate the cell viability from ability of the macrophages to metabolize MTT to purple formazan crystals, 50 μL of MTT solution (2 mg/mL – Amresco^®^) was added in each well, for 4 h, in the absence of light. DMSO was used to solubilize formazan crystals and then the absorbance was read in microplate reader (BIO-TEK Power Wave XS) at 570 nm (Mosmann, 1983). The construction of dose-response curve allowed the calculation of the cytotoxicity concentration that reduced 50% of the absorbance value when compared to the untreated NC, represented by the CC_50_. Assessing the possibility of drug-induced hemolysis, the hemolytic activity was evaluated *in vitro* from defibrinated sheep blood, commercially obtained (Laborclin; lot n° 51109038). Erythrocyte suspension at 6% was prepared by centrifugation in 2,000 × *g*, for 10 min and placed in 96-wells with the different concentrations of each compound. As positive control, it used Triton X (Synth^®^) surfactant. After 2 h at 37°C, all samples were centrifuged for 3 min, and the supernatant was harvested to verify the hemoglobin release by the absorbance measured in 540 nm.

### Drug Combination Assays

Analyzing the combination profile of BZTS with amphotericin B and BZTS with miltefosine, the checkerboard methodology was applied to obtain the combination index (CI), according to Chou and Talalay (1984), reviewed for Zhao et al. (2010). Briefly, promastigotes, axenic and intracellular amastigotes were prepared even as previously described (see the section *In vitro* Activity against *L. amazonensis*) as well the macrophages and erythrocytes, according to the section Cytotoxicity and Hemolysis Assays. Next, decreasing concentrations of BZTS was disposed into the first microplate row and the other drug (amphotericin B or miltefosine) was placed into the first microplate column. Thus, the compounds were singly evaluated in the first row and column and the checkerboard model supported the drug combination in the other wells. Next, the parasites were added and, after incubation, as described above, it was possible to determine the IC_50_ for the association. Finding the behavior of each association, the CI was calculated from the formula:

$$\text{CI=}\left({\text{IC}}_{\text{50 compound A associated}}/{\text{IC}}_{\text{50 compound A alone}}\right)+\left({\text{IC}}_{\text{50 compound B associated}}/{\text{IC}}_{\text{50 compound B alone}}\right)$$

CI values lower, equal and greater than one indicate, respectively, synergistic, additive and antagonistic profile of association tested, according established by Chou and Talalay (1984) and Chou (2010). These results allowed to evaluate the most appropriate combination, comparing the antileishmania combination activity and the cytotoxic and hemolytic combination profile. Thus, the tests to investigate the mechanism of action were performed only from the most interesting association.

### Electron Microscopy

Scanning electron microscopy was carried out to analyze the morphologic alterations on cell surface topography. Briefly, *L. amazonensis* promastigote forms (1 × 10^6^ parasites/mL) were treated with drugs concentrations related to IC_50_ and IC_90_ of BZTS and miltefosine, alone and in association, for comparison (BZTS IC_50_: 4.16 μM; BZTS IC_90:_ 13.87 μM_;_ miltefosine IC_50:_ 20.75 μM_;_ miltefosine IC_90:_ 86.66 μM; BZTS + miltefosine IC_50:_ 1.78 μM + 6.93 μM_;_ BZTS + miltefosine IC_90_: 6.93 μM + 18.89 μM). After incubation for 72 h, at 25°C, the samples were fixed in 2.5% glutaraldehyde in 0.1 M sodium cacodylate buffer (Electron Microscopy Sciences – EMS^®^) for 24 h. It used poly-L-lysine (Electron Microscopy Sciences – EMS^®^) to promote the fixed parasites adhesion over coverslips, which were dehydrated with increasing concentration of ethanol (30, 40, 50, 60, 70, 80, 90, 95, 100% – Sigma–Aldrich^®^). The samples were critical-point-dried (Baltec SCD-030) in CO_2_ to remove any water trace, placed on appropriate stub to coat with gold alloy. The micrographs were obtained by analysis in Shimadzu SS-550 scanning electron microscope (SEM).

In order to evaluate the ultrastructure changes in the treated parasites, promastigote forms were treated and fixed according described above. These samples were post-fixed with 1% OsO_4_ (Electron Microscopy Sciences – EMS^®^) and 0.8% potassium ferrocyanide (Electron Microscopy Sciences – EMS^®^), in the dark, during 60 min, at room temperature. This procedure increases the contrast to link with lipids. The samples were washed in 0.1 M sodium cacodylate buffer and dehydrated in increased concentration of acetone (50, 70, 80, 90, 95, 100% – Sigma–Aldrich^®^). All acetone content was replaced by cell diffusion for EPON^TM^ epoxy resin, gradually, and after 48 h, at 60°C, the resin was polymerized. Nanometric cuts (60–70 nm) were obtained on ultramicrotome (Power Tome X RMC Products), contrasted with 5% uranyl acetate (Electron Microscopy Sciences – EMS^®^) and 2% lead citrate (Electron Microscopy Sciences – EMS^®^). Finally, the samples were analyzed by transmission electron microscope (TEM) JEOL – JEM 1400.

### Flow Cytometer Assays

All flow cytometry analysis were performed from parasites treated with isolated miltefosine and BZTS with miltefosine association, using the following concentrations related to 2 × IC_90_: miltefosine: 173.32 μM; BZTS + miltefosine: 13.86 μM + 37.78 μM. For these studies, the treated protozoa were incubated during 24 h, at 25°C.

#### Volume Analysis of *L. amazonensis* Promastigote Forms

After to treat 1 × 10^7^ promastigote forms with 20 mM actinomycin D (positive control – Sigma–Aldrich^®^), isolated miltefosine and BZTS with miltefosine, the samples were washed two times with PBS 0.01 M and incubated. Cell volume was measured on BD FACSCalibur flow cytometer and 10,000 events were analyzed by CellQuest Pro software.

#### Evaluation of Cell Membrane Integrity

*L. amazonensis* promastigote cells were prepared according item 2.7. After incubation, the samples were washed in PBS 0.01 M and incubated with 50 μL of propidium iodide (PI – Sigma–Aldrich^®^) 2 mg/mL at 25°C, for 5 min. Membrane rupture allows the entrance of PI and its binding to cell DNA. Cell membrane integrity was measured according PI fluorescence, on BD FACSCalibur flow cytometer, in which 10,000 events were evaluated by CellQuest Pro software. Membrane rupture allows the entrance of PI and its binding to cell DNA. Digitonin saponin 40 μM (Sigma–Aldrich^®^) was used as positive control and the fluorescence measurement were compared with NC fluorescence.

#### Phosphatidylserine Exposure

Promastigote suspension (1 × 10^7^) was treated with 100 μM CCCP (carbonyl cyanide 3-chlorophenylhydrazone – Sigma–Aldrich^®^) as positive control, isolated miltefosine and BZTS plus miltefosine. At the end of 24 h, the samples were washed in 0.01 M PBS and resuspended in 100 μL of binding buffer (140 mM NaCl; 5 mM CaCl_2_; 10 mM HEPES-Na; pH 7.4), followed by the addition of 5 μL of the Annexin-V-FITC conjugated (Molecular Probes^®^). After 15 min of incubation, 400 μL of binding buffer and 50 μL of PI were added. Phosphatidylserine exhibits a binding site to connect to anionic conjugated protein Annexin-V-FITC and its fluorescence can be detected (Zwaal et al., 2005). Data acquisition (10,000 events) and analysis were carried out on flow cytometer BD FACSCalibur, equipped with CellQuest Pro software. Necrosis processes were identified by PI fluorescence in the cells while apoptotic cells were labeled with Annexin-V-FITC, regardless of the PI behavior.

#### Determination of Mitochondrial Transmembrane Potential (ΔΨm)

Parasites were prepared according 2.7.1 item, including positive control. After incubation, the samples were washed with 0.9% saline and incubated for 15 min with 1 μL of rhodamine 123 solution (Rh 123 – Sigma–Aldrich^®^; 5 mg/mL ethanol), according manufacture instructions. At the same way described above, 10,000 events were evaluated on flow cytometer BD FACSCalibur and the data were analyzed by CellQuest Pro software.

### Fluorometric Assays

#### Generation of Reactive Oxygen Species (ROS)

1 × 10^7^ promastigote/mL were treated with BZTS and miltefosine isolated and associated, with the concentration related to IC_50_ and IC_90_, for 24 h, at 25°C. Parasites were counted on Neubauer chamber in order to standardize a suspension containing 1 × 10^6^ parasites in PBS 0.01 M. H_2_DCFDA (2’,7’-dichlorodihydrofluorescein diacetate – Sigma–Aldrich^®^) 10 μM were added in the absence of the light and incubated during 45 min. This non-fluorescence chemical dye is chemically reduced by intracellular esterases, generating the fluorescence compound 2’,7’- dichlorofluorescein (DFA) and indicating the presence of ROS. Fluorescence intensity was quantified on fluorometer Perkin-Elmer Victor X3, at λ_ex_ = 488 nm and λ_em_ = 530 (Shukla et al., 2012).

#### Measurement of Nitric Oxide on Promastigote Forms and Infected Macrophages

Promastigote suspension (1 × 10^7^ parasites/mL) and peritoneal macrophages infected with *L. amazonensis* were treated with BZTS and miltefosine, isolated and combined, in the IC_50_ and IC_90_ concentrations. Promastigote forms and infected macrophages were incubated at 25°C for 72 h and 34°C for 48 h, respectively. Infected macrophages were removed with the aid of a cell scraper. Promastigote cells and infected macrophage suspension were washed in 0.1 M PBS and incubated with DAF-FM DA (4,5-diaminofluorescein diacetate – Molecular Probes^®^) 1 and 2 μM, respectively, during 30 min, at 37°C (Sarkar et al., 2011). Reaction between nitric oxide (NO) and DAF–FM DA produced a fluorescent compound – DAF-2T. Fluorescence was measured on fluorometer Perkin-Elmer Victor X3, at λ_ex_ = 495 nm and λ_em_ = 515 nm.

#### Detection of Lipid Droplets Using Nile Red Dye

Parasites were prepared and standardize in accordance to item 2.8.1. Next, 5 μL of Nile Red (Sigma–Aldrich^®^) 1 mg/mL methanol were added to treated parasites and incubated during 30 min, at dark and room temperature. Fluorescence was measured on Perkin-Elmer Victor X3 fluorometer, at λ_ex_ = 485 nm e λ_em_ = 535 (Greenspan et al., 1985). Nile Red is a phenoxazone dye that fluoresces intensely at yellow–gold color in the presence of triacylglycerols, cholesterol, or cholesterol esters, considered neutral lipids (Fowler and Greenspan, 1985).

#### Quantification of Autophagy Vacuoles

*L. amazonensis* promastigote forms were prepared and standardize as described previously (item 2.8.1). In addition, 1 μL of Wortmannin (Sigma–Aldrich^®^, 1 mg/mL), a known autophagy inhibitor, was added in the treated samples (Blommaart et al., 1997). After to add 10 μL of monodansylcadaverine (MDC; Sigma–Aldrich^®^) at dark, the samples were incubated for 1 h, at 37°C, for posteriorly analysis on Perkin-Elmer Victor X3 fluorometer, at λ_ex_ = 380 nm e λ_em_ = 525 nm (Pincus et al., 1975).

### Statistical Analysis

Numerical results were expressed by means of at least three experiments ± standard deviation (SD). Non-parametric data were analyzed using one-way ANOVA test and significant difference among means were identified using Dunnett’s test. Two-way ANOVA test were used to examine non-parametric data with multiples variables. Intergroup statistical difference was evaluated by Bonferroni’s test.

## Results

### Miltefosine with BZTS Caused Synergistic Antileishmania Activity and Antagonistic Cytotoxic Effect *In vitro*

During the combination assays, isolated compounds were evaluated in order to compare these results with those obtained from association strategy. BZTS showed promising antileishmania activity and presented less toxicity for J774.A1 macrophages than medicines currently used in the treatment against leishmaniasis, such as amphotericin B and miltefosine (**Table 1**).

**Table 1:** CC_50_ and IC_50_ values accompanied by the respective standard deviations (μM ± SD) for parasites (promastigote, axenic, and intracellular amastigote) and mammalian cells (J774.A1 macrophage and erythrocytes) treated with isolated BZTS, miltefosine, and amphotericin B.

		μM ± SD		
		
		BZTS	Miltefosine	Amphotericin B
CC_50_	Erythrocytes	>500	21.45 ± 0.75	51.75 ± 4.34
	J774.A1Macrophage	87.49 ± 9.07	55.12 ± 1.97	3.74 ± 0.59
IC_50_	Promastigote	4.16 ± 0.78	20.75 ± 0.25	0.84 ± 0.19
	Axenic amastigote	8.04 ± 0.37	0.79 ± 0.48	0.43 ± 0.19
	Intracellular amastigote	6.99 ± 1.22	1.82 ± 0.20	nd


*Due to high cytotoxicity for macrophages, amphotericin B activity against intracellular amastigotes was not determined (nd).*

The cytotoxicity assays revealed an inappropriate profile for the BZTS plus amphotericin B combination, which demonstrated additive effect for macrophages J774.A1 and synergistic effect for erythrocytes. Anyway, the high toxicity hampered the tests on infected macrophages. These results suggested that the isolated drugs are at least as toxic as the combination BZTS with amphotericin B. For this reason, BZTS with miltefosine combination was considered more appropriated to continuation of the study, since it presented an interesting combination profile about cytotoxicity, with CI > 1 value, identifying antagonistic toxic effect (**Table 2**).

**Table 2:** Combination profile of BZTS with miltefosine or amphotericin B for parasites (promastigote, axenic, and intracellular amastigote) and mammalian cells (J774.A1 macrophage and erythrocytes).

	BZTS + Miltefosine		BZTS + Amphotericin B	
		
	CI	PROFILE	CI	PROFILE
Promastigote	0.73	SYNERGISTIC	0.61	SYNERGISTIC
Axenic amastigote	0.39	SYNERGISTIC	0.13	SYNERGISTIC
Intracellular amastigote	0.96	SYNERGISTIC	nd	nd
J774.A1 macrophage	6.28	ANTAGONIST	1.09	ADDITIVE
Erythrocytes	>1.00	ANTAGONIST	<0.80	SYNERGISTIC


*Combination index (CI) higher, equal, and lower than one correspond to antagonist, additive, and synergistic profile. The high cytotoxicity for macrophages did not enable that drug combination activity using amphotericin B was determined (nd) for intracellular amastigote forms.*

Both tested combinations showed synergistic effect on all evolutionary forms of *L. amazonensis*, represented by CI < 1 values. BZTS with amphotericin B presented a pronounced synergism on promastigote and axenic amastigote forms. Synergistic antiproliferative activity and antagonistic cytotoxicity were the expected results for all combinations, since the combined drugs need to be more effective on protozoa than on the mammalian cell in order to consider the strategy of drugs association advantageous for the treatment. However, only the BZTS with miltefosine combination showed synergistic effect about antileishmania activity and antagonist effect related to mammalian cells cytotoxicity.

### *Leishmania amazonensis* Promastigotes Cells Changed after Exposed to Drugs Isolated and Combined

**FIGURE 1:**
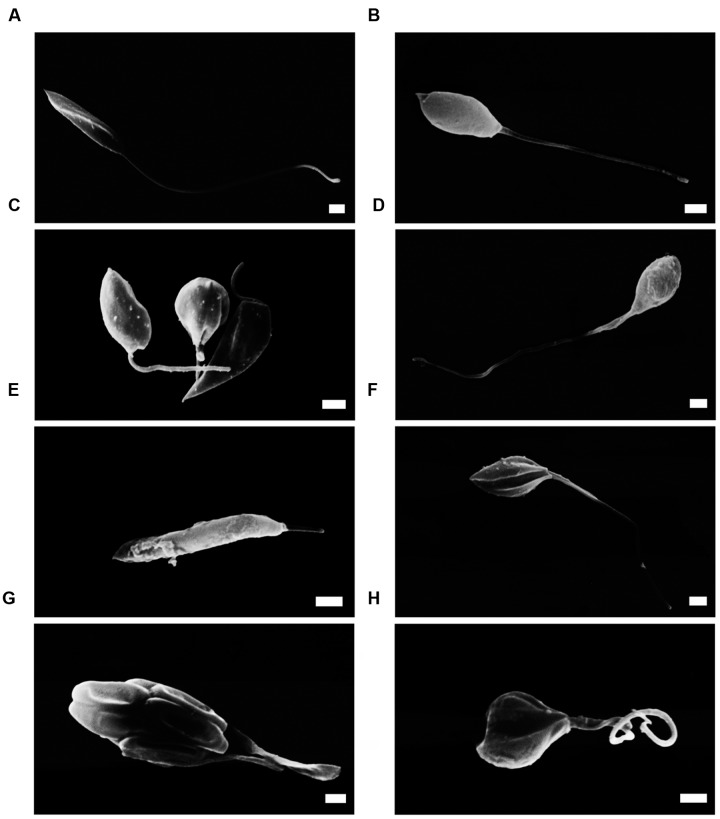
**Scanning electron microscopy (SEM) of *L. amazonensis* promastigotes.** **(A)** promastigotes control; **(B,C)** promastigotes treated with concentrations that corresponded to the BZTS IC_50_ and IC_90_, respectively; **(D,E)** morphology alterations after miltefosine IC_50_ and IC_90_ treatment, respectively; **(F)** drug combination, at IC_50_; **(G,H)** promastigote treated with IC_90_ of drug combination. Scale bar = 1 μm.

BZTS with amphotericin B showed synergistic cytotoxicity (**Table 2**). Thus, mechanisms of action assays were carried out only with BZTS plus miltefosine. By SEM, it could notice cell volume alterations and rounding of the cell body shape (**Figures 1B,C**) in comparison to the untreated promastigotes (**Figure 1A**). Differently, promastigotes exposed to miltefosine revealed changes on surface cell and reduction of the flagellum length (**Figures 1D,E**). Alterations like cell body rounding, flagellar changes and severe alterations on cellular surface were observed in the samples exposed to drug combination – BZTS + miltefosine (**Figures 1F–H**).

**FIGURE 2:**
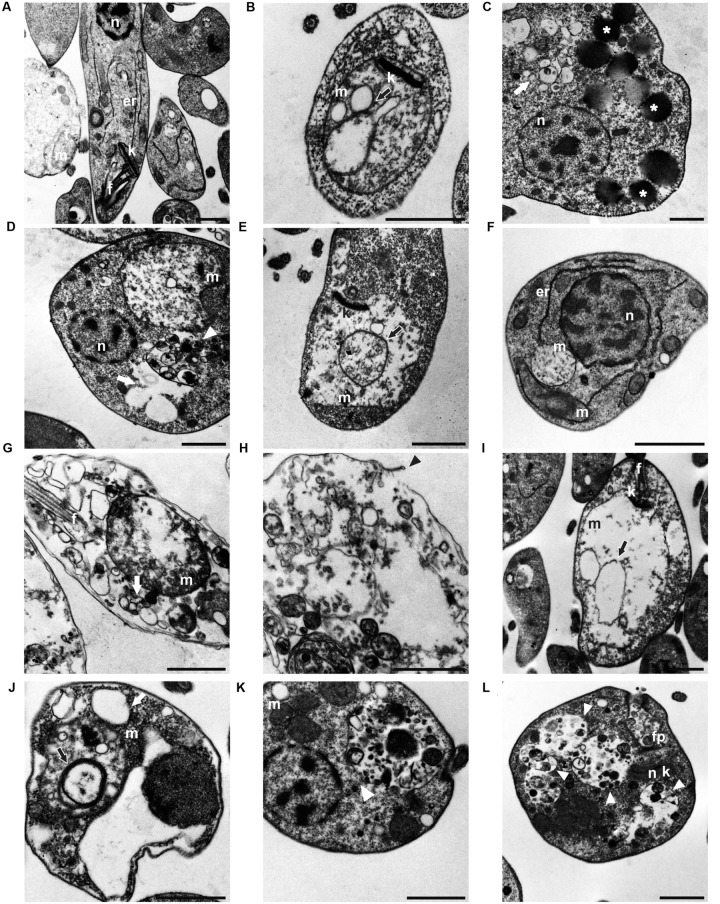
**Micrographs obtained by transmission electron microscopy (TEM) showed** **(A)** *L. amazonensis* promastigote forms with normal ultrastructure; **(B)** treated with BZTS IC_50_; **(C,D)** treated with BZTS IC_90_; **(E,F)** IC_50_ miltefosine treatment; **(G,H)** IC_90_ miltefosine treatment; **(I,J)** association between BZTS and miltefosine at the IC_50_; and **(K,L)** the IC_90_. Asterisks indicate lipid droplets on the cytoplasm; white head arrow points plasmic vacuoles; black arrows show inner mitochondrion membrane. At the micrograph **(H)**, the arrow emphasizes a rupture site of the plasmic membrane. (n) nucleus; (k) kinetoplast; (m) mitochondria, (f) flagellum, (er) endoplasmic reticulum; (Gc) Golgi complex, (fp) flagelar pocket. Scale bar = 1 μm.

Promastigotes treated with BZTS + miltefosine showed important ultrastructure changes by TEM in comparison to untreated promastigotes (**Figure 2A**). Cells treated only with BZTS showed mitochondrion alterations, like swelling and concentric membranes inner mitochondria (**Figures 2B,D**). Moreover, considerable lipid droplets were found in the cytoplasm (**Figure 2C**) even as cytoplasmic vacuolization, including autophagy vacuoles (**Figure 2D**). These findings stimulated further studies. In general, cells exposed to miltefosine exhibited similar changes (**Figures 2E–G**). Disruption of cytoplasmic membrane was observed using miltefosine (**Figure 2H**). Samples exposed to the drug combination showed similar changes to those previously mentioned. The main difference was related to the frequency of some changes, like autophagy vacuoles and mitochondrial swelling (**Figures 2I–L**).

### Synergistic Activity from BZTS Plus Miltefosine Did Not Cause Loss of Cell Membrane Integrity

**FIGURE 3:**
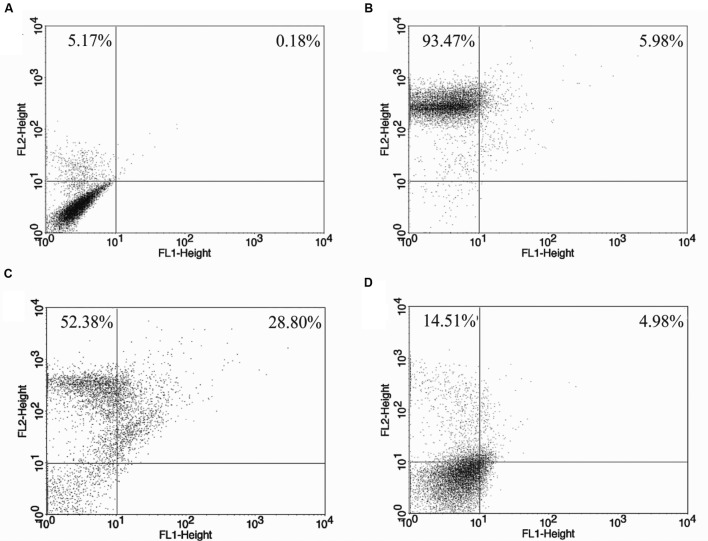
***L. amazonensis* promastigotes stained with stained propidium iodide.** **(A)** untreated control; **(B)** treated with 40 μM digitonin; **(C,D)** treated with miltefosine BZTS plus miltefosine. PI-positive cells (%) is shown in the upper quadrants.

Propidium iodide fluorescence only could be measured from cells that have lost plasma membrane integrity enabling the internalization of the specific label. PI fluorescence increased in 81.18% of promastigotes treated with miltefosine. By the other hand, the synergistic activity was not able to interfere on cell membrane integrity, represented by 19.49% of fluorescence. Digitonin, a natural surfactant used as positive control by promote cell permeabilization, exhibited 99.15% of PI fluorescence (**Figure 3**).

### Synergistic Activity from BZTS Plus Miltefosine Caused Promastigote Cells Volume Reduction and Early Exposition of Phosphatidylserine

**FIGURE 4:**
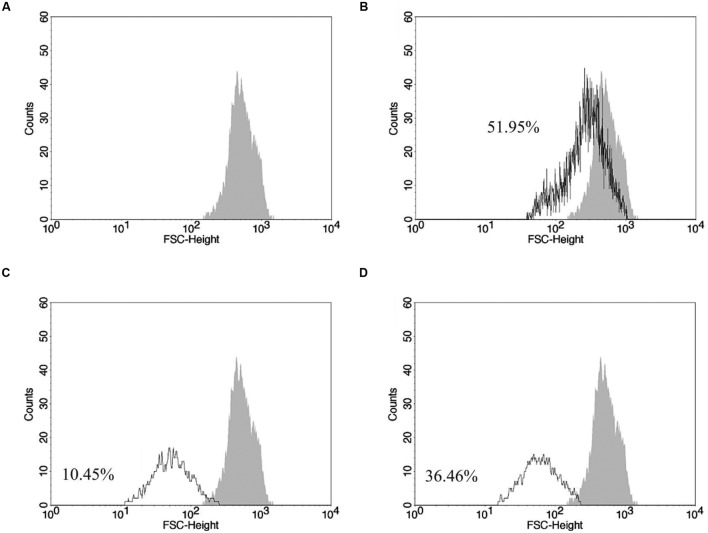
**Changes on promastigote volume analyzed by flow cytometer.** **(A)** Gray area corresponds to untreated *L. amazonensis* parasites; **(B)** actinomycin D treatment; **(C,D)** miltefosine and BZTS plus miltefosine treatment, respectively, at 173.32 μM and 13.86 μM + 37.78 μM. FSC-H was considered a function of cell size.

The results revealed decrease of cell volume after exposition to drug combination (**Figure 4**). BZTS plus miltefosine promoted volume decrease in 36.76% of the counted cells and 10.45% of promastigotes treated with isolated miltefosine presented volume reduction when compared to untreated parasites. Actinomycin D, a known apoptosis stimulator, used as positive control, causing 51.95% of cell volume reduced.

**FIGURE 5:**
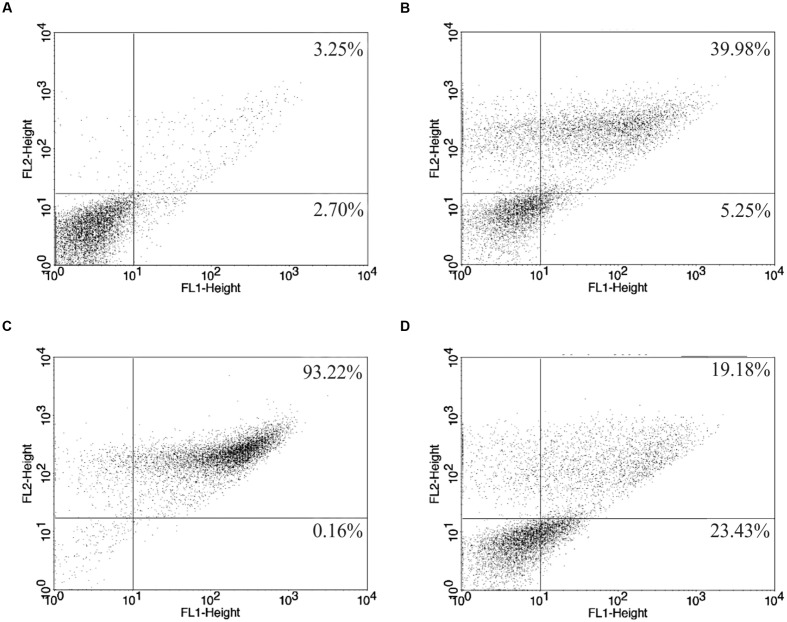
***L. amazonensis* phosphatidylserine exposure after 24 h of treatment.** **(A)** Untreated promastigote; **(B)** treated with 100 μM CCCP; **(C,D)** treated with miltefosine (173.32 μM) and drug combination (BZTS: 13.86 μM; miltefosine: 37.78 μM), respectively. Annexin-V-FITC positive cells (%) are shown in lower and upper right quadrants.

Parasites subjected to the action of the BZTS plus miltefosine demonstrated increase exposure of phosphatidylserine, as evidenced by the Annexin-V-FITC label in 23.43% of counted cells and 19.18% of the cells were considered in late apoptotic process due to present PI fluorescence. Protozoa treated with miltefosine singly resulted in 93.98% of fluorescence, indicating that most of these parasites started the late apoptotic process (**Figure 5**). Late apoptosis was also detected in 39.98% of the cells exposed to 100 μM CCCP, used in this assay as positive control. In the same sample, 5.25% of the cells showed only Annexin-V fluorescence.

### Drug Combination Altered Significantly the Mitochondrial Profile

**FIGURE 6:**
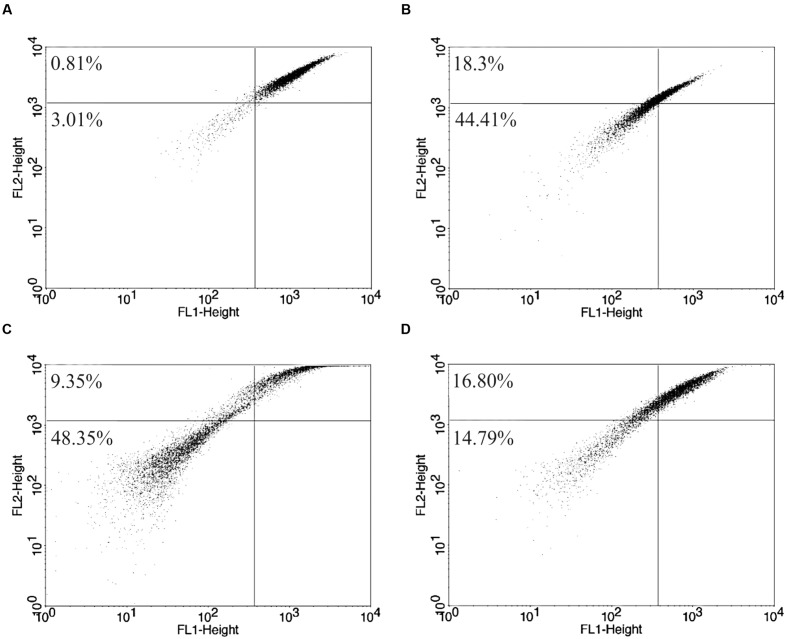
**Mitochondrial potential changes on *L. amazonensis* after 24 h of treatment.** **(A)** Untreated promastigote forms; **(B)** treated with 8 μM CCCP; **(C)** treated with 173.32 μM of isolated miltefosine and **(D)** treated with 13.86 μM of BZTS plus 37.78 μM of miltefosine. Mitochondrial membrane depolarization cells (%) are represented at upper and lower left quadrants.

Rh 123 is able to accumulate inside normal polarized mitochondrion membrane, avoiding the label escape. All treated promastigotes samples demonstrated important mitochondrial changes, according flow cytometer analysis using Rh 123 and the alterations visualized on TEM. Rh 123 fluorescence decrease indicates mitochondrial membrane depolarization and label release. Rh 123 fluorescence intensity had a strong decrease in miltefosine-treated samples and also it was observed in parasites treated with drug combination (**Figure 6**).

**FIGURE 7:**
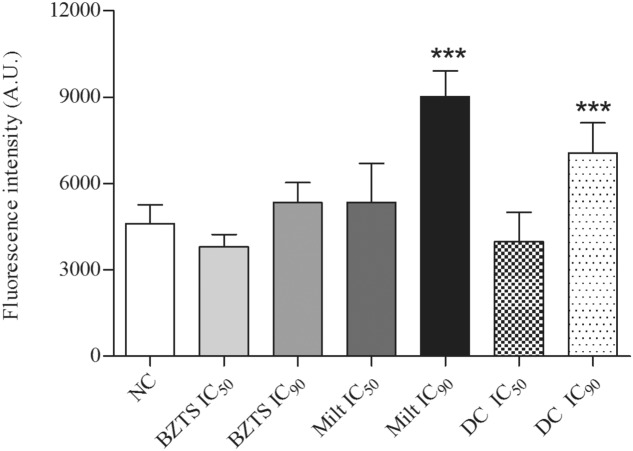
**ROS production by untreated (NC, negative control) *L. amazonensis* promastigote forms and treated with BZTS and miltefosine isolated and combined (DC, drug combination).** Detection was carried out by intensity fluorescence generated by oxidation of H2DCF-DA molecule and measured on Victor X3 (Perkin-Elmer) fluorometer. At least three independent experiments were performed. ^∗∗∗^*P* ≤ 0.001, significant difference from NC group.

Additional tests were carried out to confirm the mitochondrial changes from drug combination-treated promastigote. Data on **Figure 7** express the continuous production of reactive oxygen species (ROS) during aerobic metabolism, showing significant increase ROS development in the BZTS + miltefosine IC_90_ sample.

**FIGURE 8:**
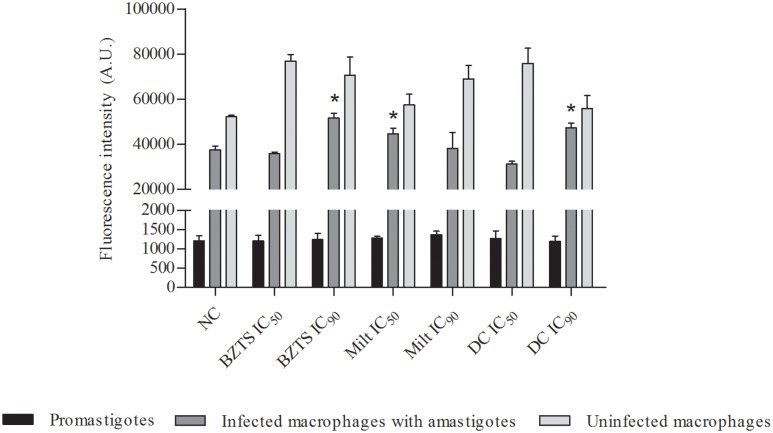
**Measure of nitric oxide production from promastigotes, macrophages infected with amastigotes and uninfected macrophages.** Cells were untreated (NC, negative control) and treated with BZTS and miltefosine alone and in combination (DC, drug combination). Intensity fluorescence emitted from DAF-2T was quantifying by Victor X3 (Perkin-Elmer) fluorometer. At least three independent experiments were analyzed. ^∗^*P* ≤ 0.05, significant difference from infected NC group.

Nitric oxide is also a highly reactive molecule and collaborates for the cellular oxidative stress development. Thus, fluorescent molecule DAF-2T was used to quantifying the NO production in promastigote and infected and non-infected macrophages. For promastigote forms, none change was observed after all treatments (**Figure 8**). Conforming expected, the NO production after contact with drug combination was relevant only for infected macrophage. Non-infected macrophages were stimulated by all treatments. After infection, it was possible to verify the NO production increased from low concentration of miltefosine. Isolated BZTS and drug combination presented this behavior only in higher concentrations.

### Synergistic Effect Increased the Development of Lipid Droplets and Autophagic Vacuole

**FIGURE 9:**
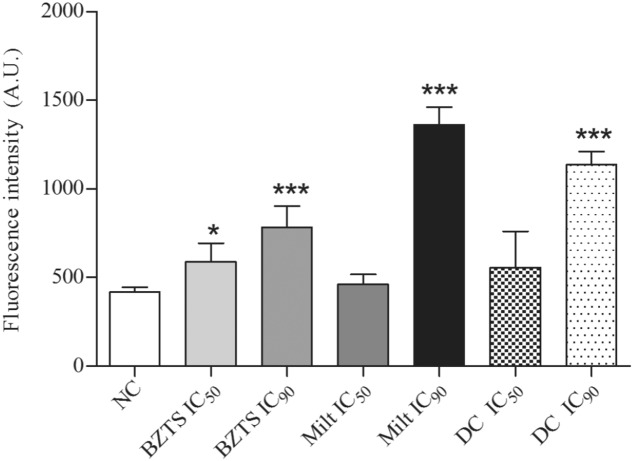
**Lipid droplets detection on *L. amazonensis* promastigotes untreated (NC) and treated with isolated and combined (DC) BZTS and miltefosine.** Nile red fluorescence was measured on Victor X3 (Perkin-Elmer) fluorometer and at least three independent experiments were performed. ^∗^*P* ≤ 0.05 and ^∗∗∗^*P* ≤ 0.001, significant difference from NC group.

Nile Red is a fluorescent dye and it has high affinity to lipids. If it is solubilized on intermediary polarity lipids (phospholipids), this dye presents red color. Or, if it is solubilized on organics solvents or neutral lipids, as triacylglycerol, its color is gold yellow (Fowler and Greenspan, 1985). These slight differences could be analyzed on fluorometer. Therefore, Nile Red was chosen to evaluate lipid droplets on promastigote cytoplasm to confirm the alterations visualized on TEM. All treated samples demonstrated the lipid droplets increase (**Figure 9**). However, only BZTS IC_50_, BZTS IC_90_, miltefosine IC_90_ and association IC_90_ showed statistical significance related to NC.

**FIGURE 10:**
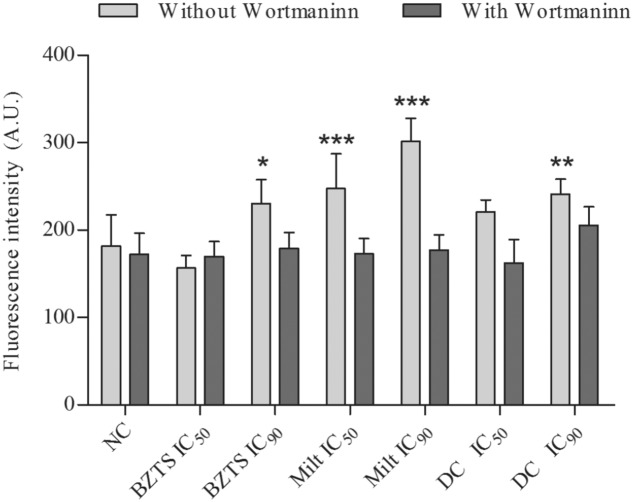
**Autophagic vacuoles quantification by monodansylcadaverine fluorescence measure.** *L. amazonensis* promastigotes were untreated (NC, negative control) or treated with isolated and combined (DC, drug combination) BZTS and miltefosine. Samples were also treated with Wortmannin, an autophagy inhibitor. Intensity fluorescence was measured on Victor X3 (Perkin-Elmer) fluorometer. ^∗^*P* ≤ 0.05, ^∗∗^*P* ≤ 0.01, and ^∗∗∗^*P* ≤ 0.001, significant difference from NC group.

Monodansylcadaverine was the label chosen to detect autophagy vacuoles after treatment with drugs alone and in association using a quantitative method. All samples treated with IC_90_ exhibited MDC fluorescence increment. In relation to MDC fluorescence from samples treated with the compounds and wortmannin, there was not statistically significant differences compared to NC or untreated parasites, suggesting that MDC was really specific to marker autophagy vacuoles generated by drugs activity (**Figure 10**).

## Discussion

Leishmaniasis is a spectrum of diseases caused by over than 20 species of *Leishmania* genus. Distinct therapeutic regimens using the same medicines – the pentavalent antimonials – are available to treat different leishmaniasis subtype. However, alterations on therapeutic regimen using a single medicine are not enough to avoid the development of resistant strains (World Health Organization, 2010).

Drug combination is an interesting and current strategy to treat diseases, which present resistance to available drugs, such as cancer and infectious diseases, including AIDS and leishmaniasis (Cullinan et al., 1994; Li et al., 2016; Trinconi et al., 2016). According to Chou (2006), different drugs acting in multiple targets or in different pathways against a single target may be more efficient to treat diseases for several reasons: drug combination increases the efficacy using lower dosage, what suggests loss of toxicity and side effects; the actuation of many targets or cell pathways slows up the resistant strains development by hinder the selection of resistant individuals to multiple mechanisms. Accordingly, these results showed that BZTS has important synergistic activity against *L. amazonensis* when combined with miltefosine or amphotericin B, both used to treat all leishmaniasis subtypes.

Drugs highly toxic and active against several infectious agents trend to show low selectivity. For this reason, wide biological activity spectrum *in vitro* from miltefosine and amphotericin B could be related to their cytotoxicity. Indeed, these isolated drugs showed this toxicity. Amphotericin B associated with BZTS kept a strong cytotoxicity on J774.A1 macrophages and erythrocytes. Intense cytotoxic effects of this combination did not allow the antileishmania activity evaluation on intracellular amastigote forms. Miltefosine associated with BZTS revealed promising synergistic antileishmania activity and antagonist cytotoxicity profile on macrophages and erythrocytes, suggesting that antileishmania activity from miltefosine associated with BZTS is not related to toxic effects on mammalian cells.

Oral treatment employing miltefosine or amphotericin B by intravenous (IV) via may cause hemolytic anemia as side effect (Cybulska et al., 1984; Queiroz et al., 2004). Intense hemolysis is the reason for miltefosine be not administrated by IV route (Zhukova et al., 2010). Miltefosine is an antineoplastic medicine that acts on kinase B protein, an important enzyme related to cell division and is responsible to cause erythrocytes lysis.’ The present results allow to conclude that miltefosine combined with BZTS softened cytotoxic and hemolytic effects concomitantly to increase antileishmania activity, characterizing the ideal for the drug association and confirm the advantages previously described by Chou (2006). Many researchers explore the possibility to combine miltefosine with other drugs, mainly interested in its proven antileishmania effect by oral route and in its still questionable safety in human’s treatment (de Morais-Teixeira et al., 2015; Trinconi et al., 2016).

Mechanism of action assays were carried out in order to compare the effects of isolated and combined drugs, making possible the investigation of cell death pathway in both cases. In all analysis in this study carried out by flow cytometry, the samples were treated with high doses (see Flow Cytometer Assays section), during only 24 h of incubation. Negative results at these circumstances could determine, for exclusion, cell pathways not reached by tested compounds. Considering this argument, it is possible to suggest the maintenance of plasma membrane integrity for drug combination treatment. Membrane rupture, swelling intracellular organelles and cell volume increase are remarkable on necrosis process (Trump et al., 1965; Laster et al., 1988). Necrosis process is not the main death mechanism for tested treatments, although occurs at the end of the cellular death. However, it seems that the treatment with high concentrations of miltefosine caused a cellular collapse, since alterations related to all death pathways were found out, what precluded the resolution of only one mechanism.

Phosphatidylserine externalization on cell surface works like a signal for phagocytes to engulf the cells and eliminate them (Segawa and Nagata, 2015). Tested drug combination and isolated BZTS presented apoptosis features in high concentration, more specifically, phosphatidylserine exposure labeled with Annexin-V-FITC. Although typical ultrastructure features of apoptosis process, like plasma membrane blebbing, chromatin condensation and formation of apoptotic bodies, were not figured out (Kerr et al., 1972), apoptosis process could not be discarded completely, mainly in drug combination, which showed positive results only Annexin-V-FITC in majority. It is evident that miltefosine induces apoptosis pathway conforming scientific literature (Dorlo et al., 2012), but the wide loss of integrity membrane resulting from treatment with high concentrations could be allowed the bind between Annexin-V and phosphatidylserine not externalized. Thus, it seems secure that high dosages even during short period are interesting to accept negative results.

Cell volume reduction observed by SEM analysis from all samples could be endorsed by flow cytometer assays for miltefosine and drug combination. Britta et al. (2014) were also reported cell volume decrease, plasma membrane integrity and positive results for Annexin-V-FITC after BZTS treatment. Apoptosis was considered the main pathway of parasites death.

Transmission electron microscope micrographs displayed several alterations after IC_50_ and IC_90_ treatment during 72 h. For the purpose of quantify these alterations, fluorometry techniques were used, conforming described at 2.8.1 item. Positive results obtained from application of lower concentration than used for flow cytometry studies, allow more confidence to propose the main cell pathways attacked.

A significant increase of autophagic vacuoles was noticed at least one tested concentration of each treatment. This short period of incubation is appropriated to study wortmannin effects, a known autophagy vacuoles inhibitor, whose stability is questionable after 20 h in culture medium (Holleran et al., 2003). Wortmannin acts on PI3k protein, which plays essential role to regulate the autophagosome maturation for promote the interaction with early and late endosomes and lysosomes (Gorvel et al., 1991; Bucci et al., 1992). MDC stain was able to consolidate the BZTS autophagy induction proposed by Britta et al. (2014). Conforming to expect, parasites treated with the tested drugs and wortmannin had lost of MDC fluorescence, suggesting that autophagic vacuoles formation was really related to drug treatment and not related to lysosomes, emphasizing the autophagic process.

Mitochondrial damages hypothesis on kinetoplastids protozoa is interesting of the selectivity point of view. This organelle is very peculiar at this protozoa family and the mitochondrial differences between protozoa and mammalian cells could be affect of distinct ways by the active compounds, checking out important specificity (Inacio et al., 2012). The results revealed mitochondrial dysfunction from treatment with drug combination by TEM analysis, mitochondrial membrane depolarization by Rho-123 and increase of ROS production by H_2_DCFDA label. Britta et al. (2014) observed mitochondrion changes only after BZTS treatment in high doses. Oxidative stress in low concentrations for drug combination and isolated miltefosine are also observed, fact that seems be decisive for the cell death. Positive results from drug combination suggest that the presence of miltefosine, even in low concentrations, enabled the BZTS antileishmania activity at shorter period.

Figarella et al. (2015) observed mitochondrial abnormalities using the same principles used in this study, with similar techniques. Ergosterone-coupled triazol molecules caused an increase of ROS production, leading to DNA damage, augmentation of autophagy and phosphatidylserine exposure, conforming reported by scientific literature for *Trypanosomatidae* family members (Figarella et al., 2006, Chowdhury et al., 2014). Jaeger and Flohé (2006) concluded that intracellular antioxidant defense of *Leishmania* spp. is totally dependent of trypanothione system, because these protozoa not present catalase and selenium-dependent glutathione peroxidase enzymes. Thus, increased oxidative stress for this protozoa is an interesting pathway to be attacked, since mammalian cells seems to have intracellular antioxidant defense system more complex (Jaeger and Flohé, 2006).

The production of reactive nitric species, represented by NO, peroxynitrite and nitrite dioxide radical, was also evaluated due to their important role on nitro-oxidative cell stress (Wiseman and Halliwell, 1996). These molecules are responsible for severe cell damages, such as DNA alterations and modulation of the genes action related to cell proliferation, differentiation and apoptosis (Dizdaroglu and Jaruga, 2012). For these reasons, the quantifying of NO was analyzed in promastigotes, firstly. Conforming the expected, there was not changes at NO level for this axenic form in all tested treatment.

By the inducible NO synthase (iNOS), NO plays really important role for defense cells, especially against intracellular parasites (Fang, 2004). NO is produced in macrophage cytoplasm and can across the cell membranes to reach parasitophorous vacuoles spread for cytoplasm or even diffuse outside the cell (Bogdan, 2001). *Leishmania* spp. is an intracellular parasite and developed, during the evolution, mechanism to evade of immune response and survival inside the host-cells. *Leishmania* intracellular amastigotes subverted the host-cell homeostasis, secreting anti-inflammatory cytokines, avoiding the production of iNOS/NO by macrophages and promoting the internalization of protozoa by recognition of phosphatidylserine molecules on the surface of the parasite plasma membrane (Olivier et al., 2005). Moreover, infected macrophages are unable to produce others pro-inflammatory cytokines necessary for the development of an effective immune response, according *in vitro* studies (Matte et al., 2001). BZTS and miltefosine, alone or in combination, induced the NO production in non-infected macrophages. After infection, there was a significant decrease of NO production, but BZTS alone and combined with miltefosine were effective to produce NO in the higher tested concentrations. The analysis also showed that NO production of miltefosine is not dose-dependent. Ponte et al. (2012) suggested that the inhibition of L-arginine oxidation, the NO precursor, could decrease NO production after contact with high miltefosine dosage.

It is still possible to suggest that mitochondrial dysfunction profile could lead to lipid metabolism changes besides supposed alteration on biosynthesis of lipid precursors generated by the treatments. Lipid bodies are dynamic intracellular organelles composed of a neutral lipid core surrounded by phospholipids and associated proteins that interact with other cell components (Murphy, 2001). These structures formation is a common process in the cell and their role is to work like fatty acid energy sources and cholesterol depots. Excessive accumulation in the cell cytoplasm is characteristic of pathological degenerative diseases in mammalian cells, like type 2 diabetes, Parkinson’s disease, some cancers and Alzheimer’s (Martin and Parton, 2005). Previous studies demonstrated increase of accumulation of lipid bodies in cytoplasm after squalene synthase inhibitor treatment and consequent inhibition of the sterol production on *L. amazonensis* (Rodrigues et al., 2008). Lipid metabolism perturbations due to inhibition of some pathway or oxidation by ROS could deposit abnormal intermediates, maybe leading to loss of the lipid original function for cell metabolism (Younce and Kolattukudy, 2012; Kathuria et al., 2014).

Autophagy is a normal cell process. It could be considered an evolutionary mechanism and an ancient adaptive function developed from eukaryotic cells. Autophagy is hazardous for collaborating to cell death, but has a beneficial role, since intend the recycling of damaged cellular components. Autophagy starts with the autophagosome formation delimited by double segregating membrane (Codogno and Meijer, 2005). Type-I programmed cell death, or autophagy, and apoptosis or type-I programmed cell death are different but related processes. In general, autophagy may be a pathway repair against toxic compounds. If cell injuries were very severe, it will occur the failure of this mechanism and consequent apoptotic cell death. In this point is understandable the several features shared between the processes, like cell volume shrinkage and mitochondrial membrane permeability. Present results may suggest that the combination therapy of drugs, which act in both pathways – autophagy and apoptosis – like BZTS and miltefosine, respectively, can generate a synergistic effect. Cell death by autophagy process have been studied from others thiosemicarbazone molecules against cancer cell lines and *Trypanosoma cruzi* (Soares et al., 2012; Carvalho et al., 2014). At kinetoplastids protozoa, autophagy seems have its great role during different life-cycle stages, according environment alterations. This allows to suggest that autophagy is a pathway that may be explored like a target against trypanosomatids infection (Brennand et al., 2012).

Reactive oxygen species and RNS are responsible for the nitro-oxidative stress and collaborate for both pathways. However, NO could modulate the autophagy on mammalian cells, working like a signal for the cell restore the homeostasis in order to inhibit the cell death (Filomeni et al., 2015). Thus, the NO produced by drug combination treatment could act protecting the macrophages cells and inducing autophagy/apoptosis death on parasites.

In summary, the association between BZTS with miltefosine showed promising *in vitro* profile against *L. amazonensis*. Antagonist cytotoxic effects could be the most attractive results, since cytotoxicity is the main problem about available leishmaniasis treatment.

Mitochondrial damage induces nitro-oxidative stress, which may promote different responses from parasites and macrophages after drug combination. Furthermore, low concentrations of the two drugs were enough to obtain a synergistic effect, what may mean that these amounts are not able to activate the cell death process on macrophages, since that host-cell has a stronger cell antioxidant defense system.

## Author Contributions

DS: Conception and design of the study, analysis and interpretation of the results, draft and writing of the manuscript. EB: Conception and execution of the experimental studies and analysis of the results, review of the manuscript. AM: Execution of the preparation of SEM and TEM samples and the image acquisition, review of the manuscript. HF and CS: Chemical synthesis and characterization of the molecule. TU-N and BD-F: Conception and design of the study. CN: Coordination of all stages of the work, conception, design and draft the study, interpretation of the results, critical review and correction of the manuscript.

## Conflict of Interest Statement

The authors declare that the research was conducted in the absence of any commercial or financial relationships that could be construed as a potential conflict of interest.

## Footnotes

1 U.S. National Institutes of Health – Clinicaltrials.com. Available online at: https://clinicaltrials.gov/ct2/results?term=triapine&Search=Search. Accessed March 10, 2016.
